# Prior information guided auto-segmentation of clinical target volume of tumor bed in postoperative breast cancer radiotherapy

**DOI:** 10.1186/s13014-023-02355-9

**Published:** 2023-10-15

**Authors:** Xin Xie, Yuchun Song, Feng Ye, Shulian Wang, Hui Yan, Xinming Zhao, Jianrong Dai

**Affiliations:** 1 Department of Radiation Oncology, National Cancer Center/National Clinical Research Center for Cancer/Cancer Hospital, Chinese Academy of Medical Sciences and Peking Union Medical College, No 17, Panjiayuan Nanli, Chaoyang District, Beijing, 100021 China; 2 Department of Diagnostic Radiology, National Cancer Center/National Clinical Research Center for Cancer/Cancer Hospital, Chinese Academy of Medical Sciences and Peking Union Medical College, No 17, Panjiayuan Nanli, Chaoyang District, Beijing, 100021 China; 3 Clinical Oncology School of Fujian Medical University, Fujian Cancer Hospital, No 420, Fuma Road, Jinan District, Fuzhou, 350011 China

**Keywords:** Auto-segmentation, Clinical target volume, Deformable image registration, Deep learning, Tumor bed

## Abstract

**Background:**

Accurate delineation of clinical target volume of tumor bed (CTV-TB) is important but it is also challenging due to surgical effects and soft tissue contrast. Recently a few auto-segmentation methods were developed to improve the process. However, those methods had comparatively low segmentation accuracy. In this study the prior information was introduced to aid auto-segmentation of CTV-TB based on a deep-learning model.

**Methods:**

To aid the delineation of CTV-TB, the tumor contour on preoperative CT was transformed onto postoperative CT via deformable image registration. Both original and transformed tumor contours were used for prior information in training an auto-segmentation model. Then, the CTV-TB contour on postoperative CT was predicted by the model. 110 pairs of preoperative and postoperative CT images were used with a 5-fold cross-validation strategy. The predicted contour was compared with the clinically approved contour for accuracy evaluation using dice similarity coefficient (DSC) and Hausdorff distance.

**Results:**

The average DSC of the deep-learning model with prior information was improved than the one without prior information (0.808 vs. 0.734, *P* < 0.05). The average DSC of the deep-learning model with prior information was higher than that of the traditional method (0.808 vs. 0.622, *P* < 0.05).

**Conclusions:**

The introduction of prior information in deep-learning model can improve segmentation accuracy of CTV-TB. The proposed method provided an effective way to automatically delineate CTV-TB in postoperative breast cancer radiotherapy.

**Supplementary Information:**

The online version contains supplementary material available at 10.1186/s13014-023-02355-9.

## Introduction

Breast cancer has become the most frequently diagnosed cancer. In 2020, there were 2.26 million women diagnosed with breast cancer and 0.68 million deaths globally [1]. Breast-conserving surgery followed by postoperative radiotherapy has become the established treatment procedure for early-stage breast cancer patients [2]. For postoperative breast cancer radiotherapy, it is important to accurately delineate the tumor bed and its target volume. However, the target volume delineation is susceptible to the number of surgical clips, clarity and size of seroma, inter-observer variability and other factors [3]. And because of asymmetric excision of the tumor during surgery, the uniform expansion of the resection cavity may not be proper to represent clinical target volume of tumor bed (CTV-TB) [4]. In general, the target volume of breast tumor bed is manually delineated by radiation oncologists in current clinical practice. However, as mentioned above, manual delineation is affected by many factors. Overall it is time-consuming and labor intensive. And there exists obvious inter-observer variability [5].

Given the intrinsic characteristics, there are several difficulties in segmenting tumor bed and its target volume for postoperative breast cancer radiotherapy. First, the contrast of soft tissue on CT image is relatively low. And the high-density marker (lead wires and titanium clips) would possibly cause metal artifacts, which compromised the image quality to some extent. Second, the contrast between tumor bed and surrounding normal breast tissue is low. The density within the region of tumor bed is close to soft tissue on the whole. Third, the size, shape and location of tumor bed varied considerably from patient to patient.

Deep learning models were popularly used in automatic segmentation of medical image [6, 7]. For postoperative breast cancer radiotherapy, there have been several researches in auto-segmenting whole breast CTV and organs at risk (OARs) [8–10]. However, there are few models developed for segmenting tumor bed and its CTV-TB due to its intrinsic complexity. Dai et al. employed a 3D U-Net to segment tumor bed, whole breast CTV and several organs at risk (OAR) on planning CT and CBCT-generated synthetic CT [11]. The results showed that the tumor bed on synthetic CT was obviously larger than the one manually contoured by physicians. The DSC (0.63 ± 0.08) was lower compared to those achieved in general medical image segmentation tasks. Kazemimoghadam proposed a saliency-based deep learning method for segmenting tumor bed [12]. It incorporated the salient information provided by titanium clip into the deep-learning model. The DSC (0.76 ± 0.03) was slightly better than that of Dai’s method but still lower in general.

Motivated by Kazemimoghadam’s method which encoded locations of titanium clips and salient regions in the deep-learning model, we proposed a method to incorporate tumor location information into the deep-learning model for segmenting CTV-TB on postoperative CT. The tumor contour on preoperative CT and its transformed contour on postoperative CT both provided prior information in searching for the potential location of CTV-TB. The rest of paper was organized as follows. In methods section, the delineation of CTV-TB and generation of prior information were first introduced. Then, the scheme of model learning and predicting was explained in detail. In results section, the effect of prior information and the model performance were evaluated. Finally, the advantages and disadvantages of the proposed method were discussed, and the future work was prospected.

## Methods

### Patient dataset

110 left-sided breast cancer patient undergone breast-conserving surgery (BCS) and eligible for whole breast irradiation (WBI) plus boost irradiation were enrolled in this study. The median age of patients was 50 years (range, 44–59 years), and the pathological diagnosis was all invasive ductal carcinoma with a stage of T1-T2N0M0. No patient received oncoplastic surgery. All patients underwent a lumpectomy with sentinel lymph node dissection. Tumor-negative margins were ensured during a single operation. Equal or more than 5 surgical clips were used to mark the boundaries of the lumpectomy cavity. All enrolled patients had either no seroma or a seroma clarity score of ≤ 3 in the lumpectomy cavity. This study was approved by the Institutional Ethics Committee of Cancer Hospital, Chinese Academy of Medical Sciences and Peking Union Medical College. Consent was waived due to the retrospective nature of the study.

The patient dataset consisted of 110 pairs of preoperative and postoperative CTs, which were acquired in the supine position. The preoperative CT was acquired averagely one week before surgery. They were reconstructed with dimensions of 512 × 512, slice thickness of 5.0 mm, and pixel size of 0.68–0.94 mm. The postoperative CT was acquired averagely 10 weeks after surgery and used for the purpose of radiotherapy treatment planning. They were reconstructed with dimensions of 512 × 512, slice thickness of 5.0 mm, and pixel size of 1.18–1.37 mm. All CTs were pre-processed using 3D Slicer (RRID:SCR_005619) [13, 14]. They were first resampled to an isotropic resolution of 1 × 1 × 5 mm and then cropped to dimensions of 256 × 256 × 32 around the breast's centroid [15].

### Contour delineation

**Fig. 1:**
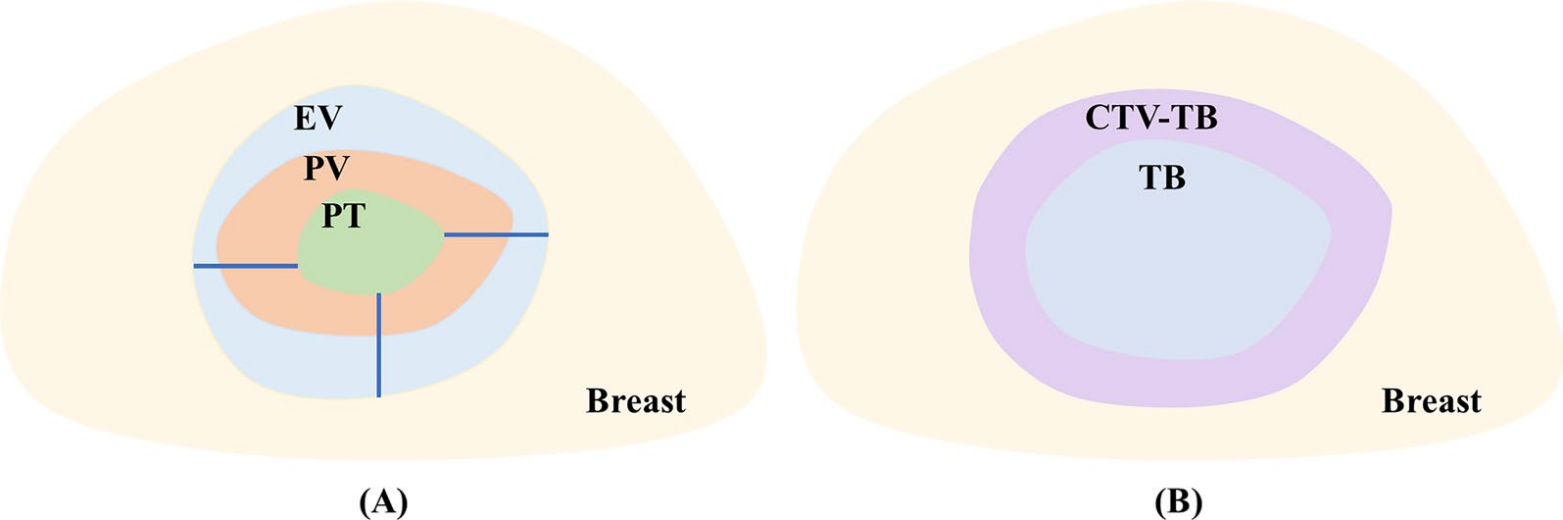
Illustration of regions of interest (ROIs) on (**A**) preoperative CT and (**B**) postoperative CT images. PT: primary tumor; PV: pathological volume; EV: excision volume; TB: tumor bed; CTV-TB: clinical target volume of tumor bed

The distribution of multiple regions of interest (ROIs) on preoperative and postoperative CTs is illustrated in Fig. 1. Before surgery, patient was CT scanned for diagnostic purpose and the location of primary tumor (PT) was manually delineated by radiation oncologist. After surgery, the volume of actually excised tissue (pathological volume, PV) was estimated by its maximum diameter in three dimensions (provided in pathological report). And the excision volume (EV) on preoperative CT image is estimated by adding a given margin to PT as shown in Fig. 1A. Different margin was tested from 1 to 3 cm. And it was found that the volume of PT with 2 cm margin is closest to the volume of PV. Thus, 2 cm margin to PT on preoperative CT image was used to represent excision volume (EV).

In a few weeks after surgery, patient was CT scanned again and proceeded to radiotherapy. As shown in Fig. 1B, clinical target volume of tumor bed (CTV-TB) is generated by adding 1 cm margin to the contour of tumor bed (TB). The margin was used to account for the subclinical lesions and potential invaded regions. In practice, the contour of TB was manually delineated by radiation oncologist according to the surgical marks and postoperative changes. Due to the poor clarity of lumpectomy cavity and relatively low soft tissue contrast, TB contouring is difficult and challenging.

### Prior information

As the location of TB is at the same place of EV before surgery, the TB contour on postoperative CT would highly correlates with the EV contour on preoperative CT. Accordingly, the TB contour plus 1 cm margin (TB_1cm_), i.e. CTV-TB, on postoperative CT would highly correlate with the EV contour plus 1 cm margin (EV_1cm_) on preoperative CT. Therefore, it would be reasonable to create virtual EV_1cm_ on postoperative CT, and used it as prior location information in searching for CTV-TB contour on postoperative CT. For reaching this goal, the deformable image registration (DIR) between preoperative and postoperative CTs was performed on Elastix (RRID:SCR_009619) [16, 17]. As a result, the deformation vector field (DVF) was obtained and used to generate the transformed EV_1cm_ (T-EV_1cm_) on postoperative CT from the EV_1cm_ on preoperative CT.

**Fig. 2:**
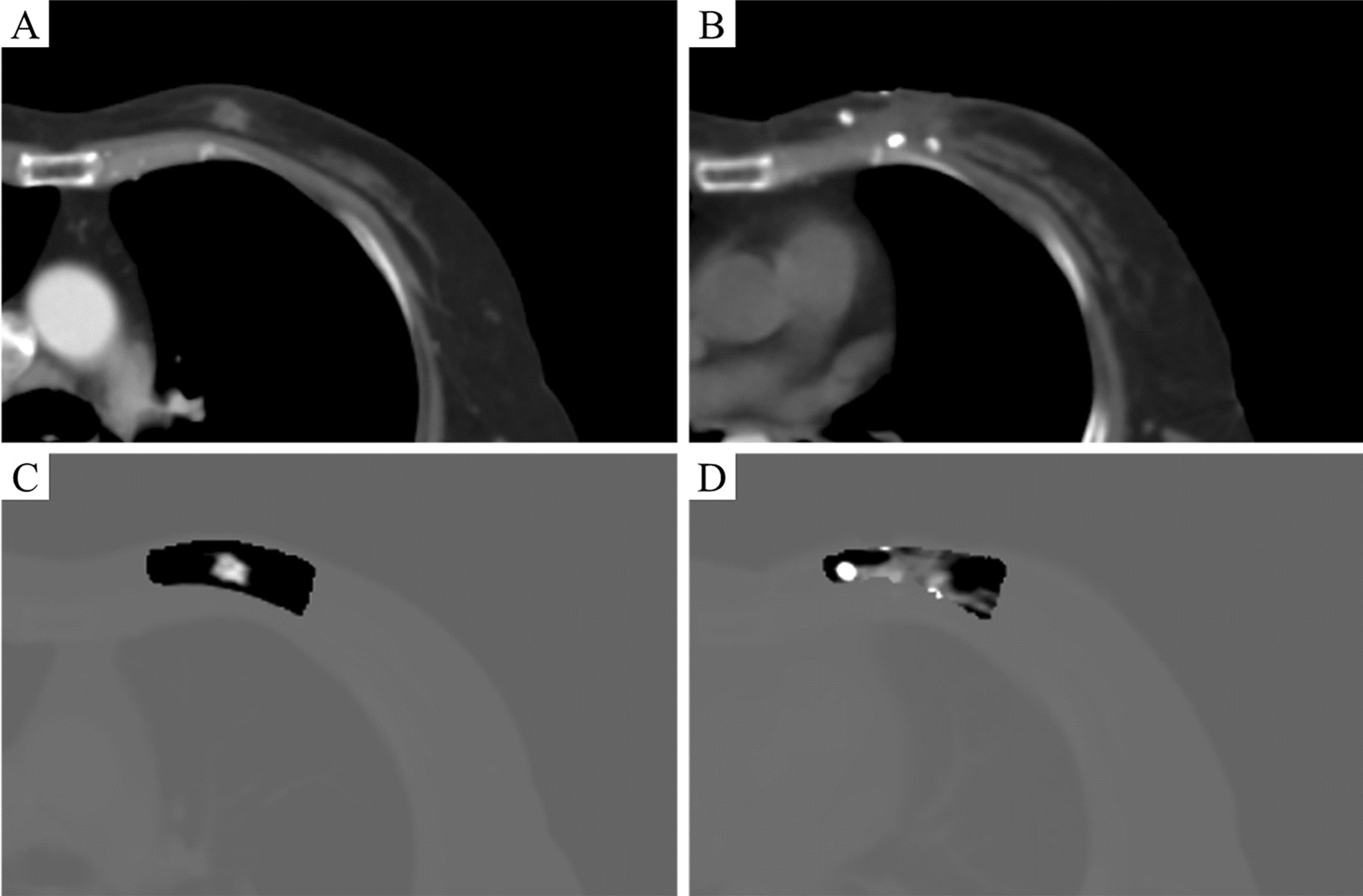
The effect of CT images before and after image enhancement. **A** The preoperative CT and **B** postoperative CT before image enhancement. **C** The preoperative CT and **D** postoperative CT after image enhancement

To enhance the effect of tumor contour on CTs, the regions of EV_1cm_ and T-EV_1cm_ were processed via image enhancement tool. In detail, the pixel values within these ROIs were multiplied by an integer number such as 25, while the pixel values outside them was multiplied by a fraction number such as 0.1. The effect of CT images before and after image enhancement is shown in Fig. 2. The preoperative and postoperative CTs before image enhancement are shown in Fig. 2A, B, while the preoperative and postoperative CTs after image enhancement are shown in Fig. 2C, D. Clearly, the intensities of tumor contours on CTs were significantly enlarged comparing with those of the surrounding tissue.

### Deep-learning model

A 3D U-Net used to solve many segmentation problems was employed in this study [18–20]. The detail of network architecture and setting was described in Additional file 1. In brief, it has an encoder part to analyze the whole image and a decoder part to produce full resolution segmentation. 3D U-Net takes 3D volume as inputs and applies 3D convolution, 3D max-pooling and 3D up-convolutional layers which has an entirely 3D architecture. In this study, there were two 3D input channels (enhanced preoperative and postoperative CT) and one 3D output channel (predicted label) in the deep learning model. A five-fold cross-validation was applied to the 110 patient dataset. One fold (22 patients) was used for testing, and the remaining four folds (88 patients) were used for training.

The weights of convolution layers are initialized by a normal distribution according to the published studies [18, 19]. The Dice similarity coefficient (DSC) was used as the loss function [19]. The Adaptive moment estimation (Adam) with batch size of 4 and weight decay of 3e−5 was used for optimization [21]. The initial learning rate was set as 0.0005, the learning rate drop factor as 0.95, and the validation frequency as 20. The network was implemented with Matlab (version 2020a) (MathWorks, Natick, MA 01760) and trained with maximal 50 epochs. The test was performed on a workstation equipped with one NVIDIA Geforce GTX 1080 TI GPU.

### Auto-segmentation of CTV-TB

**Fig. 3:**
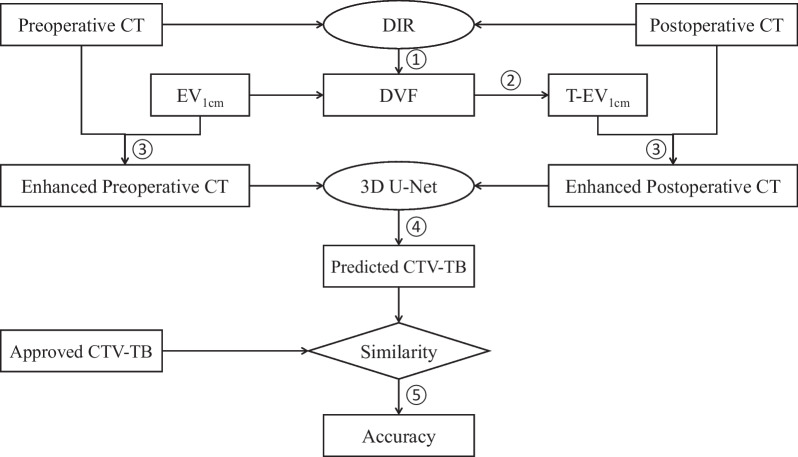
The workflow of auto-segmentation of CTV-TB on postoperative CT image

The overall workflow for segmenting CTV-TB on postoperative CT is shown in Fig. 3 and the main steps are labeled by numbers. (1) Both preoperative and postoperative CTs were registered by DIR. As a result, the DVF was obtained. (2) T-EV_1cm_ on postoperative CT was generated by deforming EV_1cm_ on preoperative CT via the obtained DVF. (3) Both EV_1cm_ and T-EV_1cm_ were processed by image enhancement tool and the resulting 3D images were fed into the deep-learning model. (4) The CTV-TB contour on postoperative CT was predicted by the deep learning model. (5) The similarity between the predicted and clinically approved CTV-TB contours was evaluated.

### Evaluations

The DSC and Hausdorff distance (HD) were used to evaluate the similarity between the predicted and clinically approved contours of CTV-TB on postoperative CT. The DSC is defined as follows [22]:

1 $${\text{DSC}}\left( {{\text{A}},{\text{B}}} \right) = \frac{{2\left| {{\text{A}} \cap {\text{B}}} \right|}}{{\left| {\text{A}} \right| + \left| {\text{B}} \right|}}$$

where *A* is the clinically approved CTV-TB contour manually delineated by the radiation oncologist and *B* is the predicted CTV-TB contour by the model. *A* ∩ *B* is the volume that *A* and *B* have in common. The DSC results in values between 0 and 1, where 0 represents no intersection and 1 reflects perfect overlap. The HD is defined as [23]:

2 $${\text{HD}}\left( {{\text{A}},{\text{B}}} \right) = {\text{max}}\left( {{\text{h}}\left( {{\text{A}},{\text{B}}} \right),{\text{h}}\left( {{\text{B}},{\text{A}}} \right)} \right)$$

where

3 $${\text{h}}\left( {{\text{A}},{\text{B}}} \right) = \mathop {\max }\limits_{{{\text{a}} \in {\text{A}}}} \left( {\mathop {\min }\limits_{{{\text{b}} \in {\text{B}}}} \parallel{\text{a}} - {\text{b}}} \parallel\right)$$

and $\left\| \cdot \right\|$ is some underlying norm on the points of *A* and *B* (e.g., the L_2_ or Euclidean norm). ${\text{h}}\left( {{\text{A}},{\text{B}}} \right)$ identify the point *a* $\in$ *A* that is farthest from any point of *B* and measures the distance from *a* to its nearest neighbor in *B*. The Hausdorff distance ${\text{HD}}\left( {{\text{A}},{\text{B}}} \right)$ is the maximum of ${\text{h}}\left( {{\text{A}},{\text{B}}} \right)$ and ${\text{h}}\left( {{\text{B}},{\text{A}}} \right)$ and measures the largest degree of mismatch between *A* and *B*. The overlap between *A* and *B* increases with smaller ${\text{HD}}\left( {{\text{A}},{\text{B}}} \right)$.

The performance of the deep-learning model with prior information (using Fig. 2C, D as inputs) was compared with the same 3D U-Net without prior information (using Fig. 2B, C as inputs). This will investigate the effect of prior information on segmentation accuracy of the deep-learning model. Five-fold cross-validation was used to tune the hyperparameters and the testing data were used to evaluate the performance of the final models. In addition, the performance of the traditional gray-level threshold method was also investigated. The gray-level threshold method partitions the gray levels in an image into two classes: those below a user-defined threshold and those above. In our study, CT values above threshold (40 HU) within the breast region were auto-segmented as CTV-TB contour. For statistical analysis, the paired t-test was performed if the data were normally distributed. Otherwise, the Wilcoxon Signed-Rank Test for Paired Samples (non-parametric test) was performed. A level of *P* < 0.05 was considered statistically significant. All statistical analyses were performed in R Project for Statistical Computing (RRID:SCR_001905) (version 3.6.3).

## Results

The training time for 3D U-Net was approximately 30 h, while the prediction time was 20 s per patient. In this binary segmentation, each pixel is labeled as CTV-TB or non-CTV-TB. The average values of DSC (mean ± standard deviation) were 0.808 ± 0.065 and 0.734 ± 0.085 for the deep-learning models with and without prior information. Comparatively, the average value of DSC (mean ± standard deviation) was 0.622 ± 0.090 for the traditional gray-level threshold method. For fair comparison, the average values of HD (mean ± standard deviation) were 19.254 ± 6.012 and 47.975 ± 22.214 for the deep-learning models with and without prior information. Comparatively, the average value of HD (mean ± standard deviation) was 60.512 ± 28.145 for the traditional gray-level threshold method.

The results of Shapiro–Wilk normality test confirmed that the data were normally distributed, so the paired t-test was used. The difference of average DSC and HD resulting from the deep-learning models with and without prior information was statistically significant (0.808 vs. 0.734, *P* = 0.0014 < 0.05; 19.254 vs. 47.975, *P* = 0.002 < 0.05). Besides, the difference between the deep-learning models with prior information and the traditional gray-level threshold method were statistically significant (0.808 vs. 0.622, *P* = 0.0005 < 0.05; 19.254 vs. 60.512, *P* = 0.001 < 0.05).

**Fig. 4:**
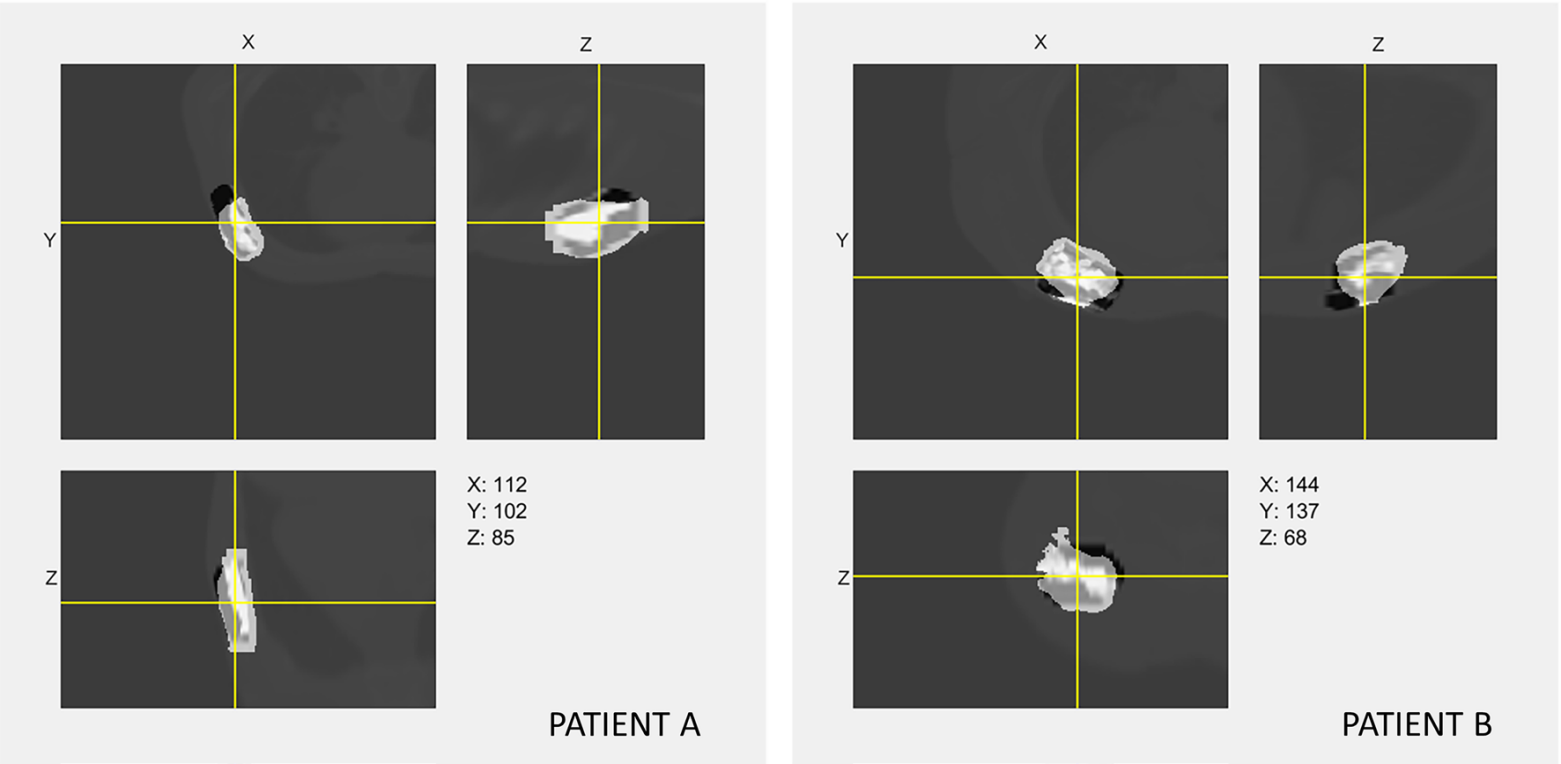
The segmentation results of prior information guided deep-learning model for two representative patients (**A**, **B**). The predicted labels are shown in white, and the ground truths are shown in black

As shown in Fig. 4, the segmentation results of prior information guided deep-learning model are displayed in three orthogonal views. The predicted labels of CTV-TB were overlaid on postoperative CT images with ground-truth (clinically approved) labels, where the predicted labels in white and the ground truths in black. It showed that the majority of both contours were similar. The predicted contour had smoother boundary than the contour which was manually delineated by radiation oncologist.

## Discussion

In this study, a prior information guided deep-learning model was developed to automatically segment CTV-TB from postoperative CT. The results showed that the introduction of prior information succeeded in identifying low-contrast CTV-TB from surrounding normal tissue on postoperative CT. This improvement would be attributed to the introduction of EV_1cm_ on preoperative CT and T-EV_1cm_ on postoperative CT, which bring prior information about the approximate CTV-TB contour and set a good starting point for the deep learning model. Besides, the DSC and HD of the transformed T-EV_1cm_ contour and clinically approved CTV-TB contour were 0.551 ± 0.110 and 49.875 ± 23.514. It is worth noting that intensity-based DIR method is challenged due to the large deformations and non-correspondence caused by tumor resection and clip insertion. Thus the introduction of T-EV_1cm_ on postoperative CT just provide the approximate location of CTV-TB contour.

3D U-Net was previously used in segmenting tumor bed on CBCT-generated synthetic CT and the DSC was lower [11]. Later, Kazemimoghadam incorporated the salient information provided by titanium clip into the U-Net model for tumor bed segmentation. The DSC was improved but limited [12]. In our study the DSC was further improved. The improved DSC would be attributed to the introduction of prior information, which limited the searching range for potential CTV-TB contour on postoperative CT. It should be noted that both Kazemimoghadam’s and our methods incorporated prior information in segmentation model to aid the searching of final target.

There are certain limitations of this study. First, the training set is small, which requires massive cross-validation to ensure the stability of learning model. More data will be collected in the future to make the model more robust. Second, the intensity-based DIR was used to generate DVF for transforming tumor contour onto postoperative CT. More advanced DIR methods would be investigated and adopted for future study. Third, only CT image was used for the input of deep learning model. It would be more interesting to include other image modalities such as CBCT, Ultrasound and MRI. With these inputs the segmentation accuracy of the deep learning model would be further improved. Fourth, manual contouring on preoperative CT image was still needed. In the future, auto-segmentation method would be investigated to further improve efficiency.

## Conclusions

Incorporating prior information of tumor location into deep learning model improved the segmentation accuracy of CTV-TB contour on postoperative CT. The tumor contours on both preoperative and postoperative CT provided the approximate CTV-TB contour, which facilitated the subsequent searching by the deep learning model. The proposed method demonstrated an effective way in auto-segmentation of CTV-TB in postoperative breast cancer radiotherapy.

### Supplementary Information

**Additional file 1:** The detail of network architecture and setting.

## Data Availability

The datasets used and/or analysed during the current study are available from the corresponding author on reasonable request.

## Abbreviations

CTV-TB: Clinical target volume of tumor bed
TB: Tumor bed
CT: Computed tomography
DIR: Deformable image registration
DSC: Dice similarity coefficient
HD: Hausdorff distance
OARs: Organs at risk
BCS: Breast-conserving surgery
WBI: Whole breast irradiation
ROIs: Regions of interest
PT: Primary tumor
PV: Pathological volume
EV: Excision volume
DVF: Deformation vector field
Adam: Adaptive moment estimation
