# Supplementary material for: Prior information guided auto-segmentation of clinical target volume of tumor bed in postoperative breast cancer radiotherapy
Source: Radiat Oncol. 2023 Oct 15;18:170. doi: 10.1186/s13014-023-02355-9 (PMC10577969; doi:10.1186/s13014-023-02355-9)
Supplement: Supplementary file 1 — Additional file 1: The detail of network architecture and setting. [file 13014_2023_2355_MOESM1_ESM.docx]

**Supplementary material**

**Additional file 1**

3D U-Net was introduced shortly after U-Net to process volumetric data which is abundant in medical data analysis. In this study, a 3D U-Net architecture previously used for brain tumor segmentation is employed as shown in Figure A1 (1). The initial series of convolutional layers were interspersed with max pooling layers and successively decreased the input image's resolution from 132 to 9 in the encoding process (left part of Figure A1). These layers were followed by a series of convolutional layers interspersed with upsampling operators, successively increasing the input image's resolution from 9 to 132 in the decoding process (right part of Figure A1). A batch normalization layer was introduced before each rectified linear unit (ReLU) layer. In the original U-Net implementation, all convolution, max pooling, and upsampling operations were carried out in 2D (2). This was later extended to a 3D U-Net by Çiçek (1).


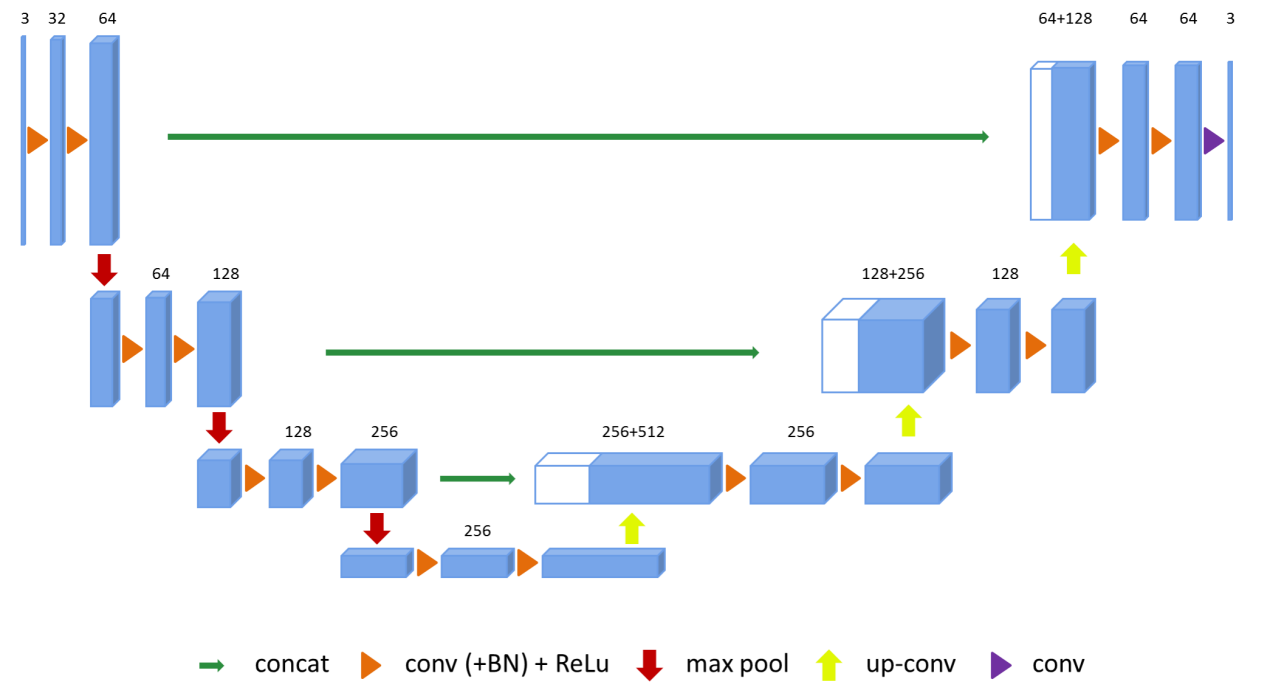


**Figure A1** The 3D U-Net architecture. Blue boxes represent feature maps. The number of channels is denoted above each feature map.

The 3D U-Net was trained on image patches, and the test patches were finally stitched into a complete segmented test volume through an overlap-tile strategy. A random patch extraction datastore that contains the training image and pixel label data was used to feed the training data to the network. The patch size was 132×132×132 voxels, and 16 randomly positioned patches were extracted from each pair of volumes and labels during training. For prediction, the overlap-tile strategy was used to predict the labels for each test volume, which was then padded to make the input size a multiple of the network's output size and compensated for the effects of valid convolution. The overlap-tile algorithm selected overlapping patches, predicted the labels for each patch, and then recombined the patches, which avoids border artifacts using the valid part of the convolution in the neural network (2).

Like the standard U-Net, the deep-learning network used in this study has an encoding path and a decoding path. In the encoding path, each layer contains two 3×3×3 convolutions, each followed by a ReLu, and then a 2×2×2 max pooling with strides of two in each dimension. In the decoding path, each layer consists of an up-convolution of 2×2×2 by strides of two in each dimension, followed by two 3×3×3 convolutions, each followed by a ReLu. Shortcut connections from layers of equal resolution in the analysis path provide the essential high-resolution features to the synthesis path. In the last layer, a 1×1×1 convolution reduces the number of output channels to the number of labels which was 2 in our case. The architecture has 1.907e7 parameters in total. Bottlenecks could be avoided by doubling the number of channels already before max pooling and this scheme was adopted in the synthesis path (3).

**Reference**

1. Çiçek Ö, Abdulkadir A, Lienkamp S, Brox T, Ronneberger O. 3D U-Net: Learning Dense Volumetric Segmentation from Sparse Annotation. 2016.

2. Ronneberger O, Fischer P, Brox T. U-Net: Convolutional Networks for Biomedical Image Segmentation. 2015.

3. Szegedy C, Vanhoucke V, Ioffe S, Shlens J, Wojna Z. Rethinking the Inception Architecture for Computer Vision. 2015.
